# Congenital Cytomegalovirus (cCMV) Infection as a Leading Cause of Pediatric Hearing Loss: Review

**DOI:** 10.3390/children12050613

**Published:** 2025-05-08

**Authors:** Amber Cradeur, Aaron Jackson, Erin Ware, Torrey L. Fourrier, Gauri Mankekar

**Affiliations:** 1 School of Medicine, Louisiana State University Health Shreveport, 1501, King’s Highway, Shreveport, LA 71103, USA; alc007@lsuhs.edu (A.C.); ajj001@lsuhs.edu (A.J.); 2 Library, Louisiana State University Health Shreveport, 1501, King’s Highway, Shreveport, LA 71103, USA; eew001@lsuhs.edu; 3 Department of Otolaryngology-Head and Neck Surgery, Louisiana State University Health Shreveport, 1501, King’s Highway, Shreveport, LA 71103, USA; torrey.fourrier@lsuhs.edu

**Keywords:** congenital cytomegalovirus, pediatric hearing loss, systematic review, meta-analysis, diagnostic accuracy

## Abstract

**Background/Objectives:** Congenital cytomegalovirus (cCMV) infection is a significant cause of pediatric hearing loss. However, the prevalence and characteristics of cCMV-related hearing loss remain unclear. **Methods**: A comprehensive search of major databases (PubMed, Scopus, and Web of Science) was conducted to identify studies and reviews published between 2000 and 2024 that investigated cCMV infection, testing for cCMV, and pediatric hearing loss. Studies were included if they reported on the prevalence, characteristics, current policies, and recommendations for universal cCMV testing in newborns. **Results**: This review highlights key issues: cCMV is a significant and treatable cause of pediatric hearing loss; most cCMV-related hearing loss cases presented with bilateral and profound hearing impairment; and cCMV testing is performed only for babies who fail newborn hearing screening. **Conclusions**: This narrative review highlights the significant association between cCMV infection and pediatric hearing loss. Currently, targeted cCMV testing is recommended for newborns who fail newborn hearing screening. Universal cCMV testing may facilitate early diagnosis and directed intervention and could be cost-effective in the long run.

## 1. Introduction

Congenital cytomegalovirus (cCMV) infection is the leading non-genetic cause of hearing loss in the pediatric population worldwide. While most infected newborns are asymptomatic, a significant number will either be born with hearing loss or develop it later in life. The early initiation of antiviral therapy, such as ganciclovir or valganciclovir, has been reported to improve audiological and neurodevelopmental outcomes [1,2]. The European Congenital Infection Initiative (ECCI) consensus statement recommends starting valganciclovir as soon as possible for newborns with isolated sensorineural hearing loss (SNHL), as well as those with CNS symptoms [3]. However, this is controversial. A recent systematic review did not find an adequate benefit of valganciclovir on audiological outcomes in children with isolated hearing loss, late-onset hearing loss, or asymptomatic CMV [4].

Many states and hospital programs currently implement a targeted approach to cCMV screening, which involves further evaluation when a newborn does not pass their universal neonatal hearing screening. However, several studies criticize this screening method for its inadequacy in properly identifying all infected infants who are at risk of complications. While there has been significant advancement in the early detection of cCMV within recent years, secondary to the implementation of newborn hearing screening, there is still an opportunity to improve screening protocols and methodology. The current literature describes the present state of cCMV screening and detection, details the inadequacies, and largely supports the notion of implementing universal neonatal screening for cCMV.

**Figure 1:**
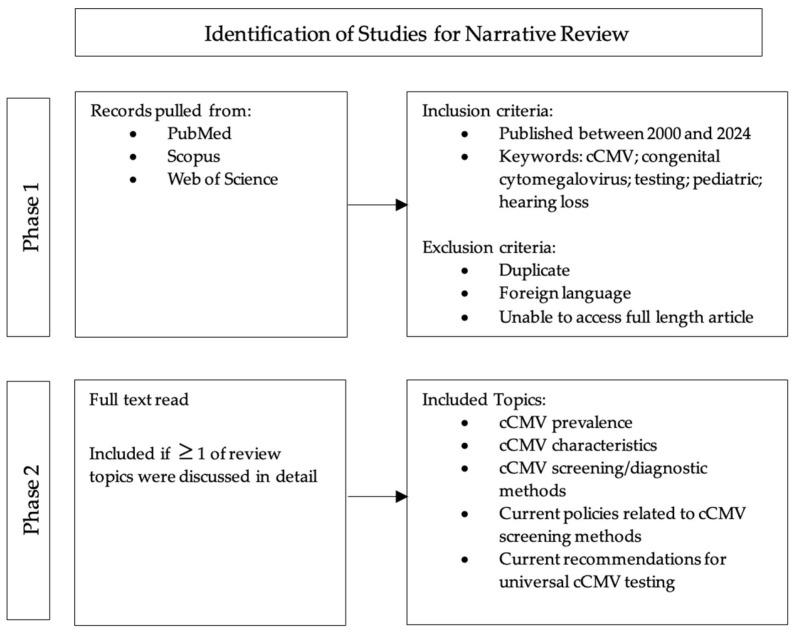
Literature search methods.

To accurately capture the prevalence, clinical presentation, and current state of cCMV screening and detection, various phases and criteria were used in the literature search for this narrative review (Figure 1). The first phase of the literature review involved an extensive search of major databases (PubMed, Scopus, and Web of Science) to identify studies published between 2000 and 2024 that investigated cCMV infection, diagnostic testing for cCMV, and pediatric hearing loss. Words such as congenital cytomegalovirus, cCMV, screening, and hearing loss were utilized as indicators for article/study inclusion. Abstracts were subsequently reviewed to ensure the accessibility of the article and were excluded if not written in the English language or if the full text was unavailable. The second phase began by reading each article/study that had passed the initial inspection and inclusion criteria. Items that reported the prevalence, characteristics, diagnostic methods, current policies, and/or recommendations for universal cCMV testing in newborns were finally included. Once the final publications were identified, the information and data were compiled, presented, and analyzed by the research team.

This narrative review aimed to highlight the prevalence of cCMV, particularly in the United States; provide insights into the clinical manifestations of cCMV; and summarize the ongoing discussions regarding CMV screening recommendations.

## 2. Prevalence of cCMV

The impact of cCMV is significant, as it is the leading non-genetic cause of sensorineural hearing loss and a major infectious contributor to neurodevelopmental disabilities in children [5]. Thus, understanding the prevalence of cCMV, particularly the demographics that it affects, is crucial for guiding public health strategies in the prevention and management of at-risk populations.

Globally, cCMV infection has a prevalence at birth varying between 0.2% and 1% (depending on the country’s maternal seroprevalence rates) [6]. In the US, this translates to approximately 20,000 to 30,000 infants infected each year [7]. In other developed countries like Canada, those in Western Europe, and Australia, the prevalence of congenital CMV infection is similar, at 0.5% to 0.7% of all live births [8]. Within developing countries, cCMV has an estimated incidence of 0.6–0.7% of all live births [5].

A recent study investigated the impact of advanced maternal age and intrauterine growth restriction on cCMV prevalence. A cohort study from China found that 75 out of 10,993 screened newborns tested positive for cCMV, resulting in an overall prevalence of 0.7%, supporting values found in other studies [9]. Of note, the prevalence of cCMV infections declined with increasing maternal age and increased in preterm infants and those with intrauterine growth limitation, compared to full-term infants and those without these conditions [9].

Some sources cite a disparity in CMV prevalence by maternal race, ethnicity, and geographic location. A recent investigation into previously published cCMV newborn screening studies in the United States found that the adjusted prevalence of cCMV infection, accounting for maternal race and ethnicity, was estimated to be between 4.6 and 4.7 cases per 1000 live births on a national level and to range between 3.9 and 6.5 cases per 1000 across states from 2018 to 2022 [10]. Notably, infants born to non-Hispanic Black mothers had the highest prevalence at 9.3 cases (with a range of 8.2 to 10.5 per 1000), followed by non-Hispanic American Indian and Alaska Native mothers at 8.5 cases (with a range of 2.1 to 33.2 per 1000) [10]. Additionally, the annual prevalence of cCMV infection was consistently highest in the southern states and Alaska [10]. These findings indicate that states with a greater proportion of racial and ethnic minorities have a higher prevalence of cCMV than states with predominantly White populations, and they highlight the importance of cCMV surveillance at the jurisdiction level and the need for tailored and culturally relevant education and prevention strategies for those at higher risk [10].

## 3. Clinical Presentation

Congenital cytomegalovirus infections typically occur when cytomegalovirus, a member of the Herpesviridae family, is transmitted vertically, through the placenta, from mother to fetus during pregnancy [9,11]. Primary maternal infections occur when a pregnant mother first becomes infected with CMV through close contact with bodily fluids/secretions. This infection subsequently poses a higher risk of vertical transmission than non-primary maternal infections, which are characterized as the reactivation of latent CMV infection or reinfection with a new strain of CMV during pregnancy [7,9,12]. Additionally, cCMV infections resulting from primary maternal infections are more likely to present with symptoms at birth, even when fetal imaging is normal, compared to non-primary maternal infections [9,13].

The presentation of cCMV may be categorized into symptomatic and asymptomatic. Most infected infants present with no clinical features, and the long-term prognosis for those with clinically inapparent infections is excellent [11]. Only about 11% of live infants born with cCMV are symptomatic and present with abnormal clinical findings at birth, with 25% of those infected infants developing sequelae by the age of 2 years [7,14,15]. For those with symptomatic cCMV infections at birth, clinical manifestations can vary and include small for gestational age, intrauterine growth restriction, microcephaly, blueberry muffin rash representing extramedullary hematopoiesis, hepatosplenomegaly, petechiae, thrombocytopenia, jaundice, direct hyperbilirubinemia, elevated alanine aminotransaminase, chorioretinitis, or cerebral calcifications that may be seen as hyperintense signal alterations on MRI [11,13,16,17].

Perhaps the most well-known and feared complication of cCMV is the risk of hearing impairment in the form of SNHL [14,17,18,19]. Between 20% and 65% of symptomatic infants and 6% and 15% of clinically inapparent at-birth infants will develop SNHL, with severity ranging from mild to profound in unilateral or bilateral ears [7,8,17,20]. This CMV-related hearing loss typically has a fluctuating and progressive course, sometimes resulting in children being undiagnosed until elementary age if not initially apparent at the time of newborn hearing screening [21]. Possible prognostic predictors for hearing outcomes include symptoms of cCMV at birth, failure of neonatal hearing screening, and viral load level in blood [22]. Missed opportunities for the early diagnosis and treatment of cCMV could ultimately lead to severe hearing loss or profound developmental delay [23].

While estimates of long-term sequelae are likely underestimated due to the lack of universal testing at birth and limited follow-up in clinical trials, a retrospective observational study aimed at describing the long-term outcomes of cCMV infection in children identified 61 newborns positive for CMV through a PCR of viral DNA from urine within the first 15 days of life [7,24]. Of the 61 newborns, 20 were diagnosed with sensorineural hearing loss (SNHL), 8 of whom underwent cochlear implantation [24].

## 4. cCMV Screening Methodology

With early cCMV detection being key in offering treatment and subsequently helping create positive outcomes, many studies have focused on establishing a consistently reliable, feasible, and cost-efficient method to maximize cCMV detection. Methods that have been recently studied and/or implemented in country-wide screening recommendations include perinatal antibody and avidity testing, PCR (polymerase chain reaction) testing of neonatal saliva or urine, and DBS (dried blood spot) testing.

Researchers have studied maternal CMV screening as a predictor of congenital CMV infection in newborns. IgM antibodies that are associated with acute infection can persist for months with IgG antibodies. Therefore, it is difficult to isolate the time of infection. While high IgM with high IgG suggests an infection, it does little to provide a clue as to the infection timeline. This is not a reliable practice for widespread diagnostic utility; however, other testing, such as viral avidity testing, may prove useful.

Viral avidity testing measures the affinity with which IgG antibodies bind to the virus and has been used to assess the risk of fetal transmission in infections such as toxoplasmosis, parvovirus, rubella, and CMV [12]. Reduced viral avidity in recently infected cases (12–18 weeks) acts as an indicator of increased circulating levels of the virus and the risk of fetal transmission, while high avidity indicates an improved immune response, decreased transmission, and higher titers of virus-neutralizing antibodies [12].

A recent study by Raynor et al. investigated the utility of detecting cCMV and predicting SNHL with the concept of avidity [12]. Although a promising concept, the findings showed no significant hearing loss in babies born to CMV-positive mothers, likely because most mothers had a strong immune response (high viral avidity), indicating infection before pregnancy [12]. Consequently, although viral avidity may help to determine whether an infection is recent or from the past, it may not reliably differentiate between congenital infections that pose a risk of SNHL and those that do not, limiting its usefulness in a universal screening protocol.

In neonates, when there is a high suspicion of intrauterine CMV infection, viral isolation and culture from urine or saliva is the traditional standard [14]. The high sensitivity of PCR, alongside the ease of collection and testing of saliva samples, makes this the preferred option [14]. However, the high sensitivity is also associated with a high number of false-positive tests that have been associated with saliva, and any positive saliva result should be subsequently confirmed with a urine sample [14,25]. Importantly, to detect congenital CMV infection, the sample must be collected at birth, as a diagnosis made after the first 2–3 weeks of life could be due to viral transmission through the breast milk of a seropositive mother, possibly contributing to the high rate of false positives [13]. Despite the high false-positive rate, this strategy could provide a convenient and efficient approach to detecting neonatal infection in less developed parts of the world with an otherwise reduced detection capacity [26,27]. However, there have been citations of concern with the cost of the widespread administration of such tests [28]. Targeted urine-filtered screening presents a viable option for neonatal CMV screening when used by itself or as a confirmation exam [29].

Dried blood spot (DBS) testing is a reliable, cost-effective alternative to saliva swabs or urine cultures that may be used to diagnose congenital CMV. This method may offer long-term sample storage and high accuracy (71–100% sensitivity and 99–100% specificity) [30,31]. The broad range of sensitivity offers some controversy on whether this method could dependably offer an accurate diagnosis if implemented universally [32]. However, those who support this method point to the ability of DBS PCR testing to be integrated into existing newborn screening protocols, encouraging the utility of this option for neonatal screening and diagnosis [25]. Previous studies have shown the utility of Guthrie card DBS samples, positively identifying all neonates with a cCMV infection, including those who were asymptomatic and without some degree of hearing impairment [23]. DBS screening for cCMV could be adequate in clinical practice and presents a practical, viable approach for universal mass screening [33].

Some studies have investigated the benefit of a combined, comprehensive program that integrates physiologic, genetic, and cCMV screening alongside universal newborn hearing screening [34]. By identifying at-risk infants who would otherwise go unnoticed through conventional methods, this strategy ensures early intervention, which is paramount for speech and cognitive growth and development. Additionally, obtaining etiological data early on enables personalized treatment planning, removing ambiguity from families and clinicians. By maximizing diagnostics, this strategy may also limit the loss to follow-up of children, ensuring that they have adequate care. Furthermore, the early identification of risk factors may reduce the need for costly repeated testing throughout later childhood, ultimately optimizing healthcare resources, with improved long-term outcomes among affected children. In summary, this strategy could work to enhance the early detection of hearing loss, provide etiologic insights, improve follow-up rates, and reduce long-term testing costs [34].

## 5. Targeted Neonatal Screening

According to international standards, targeted screening is currently indicated either when a newborn fails their hearing screening protocol, for example, the Transient Evoked Otoacoustic Emissions (TEOAE) assessment, or when a newborn presents with findings suggestive of cCMV infection, such as thrombocytopenia, jaundice, or microcephaly [12,15,35]. If either indication is met, a newborn will undergo more “targeted” testing, typically through the detection of viral DNA by PCR of a saliva or urine sample [35,36,37]. A neonate with confirmed cCMV and hearing loss can then be offered antiviral therapy for implementation within their first month of life [38].

Currently recommended by the American Academy of Audiology, as well as the Newborn Hearing Screening Working Group of the National Coordinating Center for the Regional Genetics Networks, targeted cCMV screening is the most implemented screening measure to detect cCMV infections in newborns [11,16,35]. Its use is seen widely in the United States, Canada, the United Kingdom, Europe, and Australia [35,39,40].

Designed as a prospective survey of birth hospitals performing early cCMV testing, the NIH ValEAR clinical trial was a study aimed at determining the positivity rate of the three main approaches: universal CMV testing, hearing-targeted CMV testing (HT-cCMV), and delayed targeted dried blood spot (DBS) testing [8]. The researchers found that the positivity rates of cCMV among the three different approaches were 0.5%, 1.5%, and 7.3%, respectively, with results consistent with cohort studies reported in the literature [8]. The largest group of infants (n = 9017) underwent HT-cCMV screening, and this group comprised the greatest number of detected CMV infections at n = 132 [8].

A recent study investigated parents’ perceptions of such testing and found that families felt as though it was feasible, acceptable, and generally a good idea to test the neonate for congenital infection [41]. Further investigation into the associated cost indicated that implementing targeted cCMV screening is both feasible and financially viable, with minimal cost differences compared to non-screening [42].

Targeted screening is widely endorsed as the standard practice, whereas universal screening, though less common, is increasingly viewed as the best approach for identifying children with cCMV [35,43].

## 6. Current State and Perceptions of Universal Neonatal Screening

Universal neonatal screening may ultimately lead to the early diagnosis of cCMV, thus catching the infection early and offering an opportunity to treat and potentially avoid a devastating and debilitating diagnosis of SNHL. This may ultimately improve both linguistic skills and cognitive development in the affected population [44]. Coupled with the proven effectiveness and increase in accessibility, the proven benefits of early identification and intervention on auditory outcomes are undoubtable [44,45]. Current international guidelines maintain recommendations for cCMV screening for infants who fail universal newborn hearing screening [38]. However, this presents a missed opportunity to accurately diagnose those who will ultimately present with late-onset hearing loss, secondary to the CMV [46,47,48]. Perhaps one of the most significant barriers to implementing universal neonatal cCMV screening is the cost. A recent study by Saito et al. comparing the direct and long-term care costs of universal and targeted screening suggested that, despite higher incremental costs, universal screening is ultimately more cost-effective than targeted screening [49].

Despite cCMV infection meeting many of the criteria for a universal screening program according to the WHO (World Health Organization), and a multitude of studies trialing such a program in hospital centers with promising results, no country has yet implemented such a program [6].

### 6.1. Within the USA

**Figure 2:**
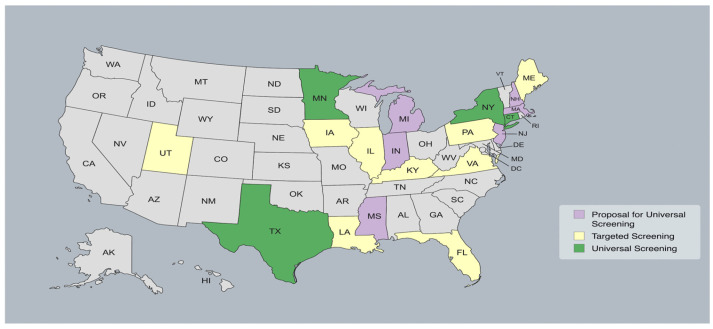
cCMV screening landscape across the United States.

The proven benefits of early identification and intervention (at <6 months of age) in terms of language outcomes and communication have confirmed the effectiveness and utility of universal cCMV neonatal screening. Further, recent studies support the greater overall cost savings and maximal opportunity to deliver targeted medical intervention to those in need [46,50,51]. The cCMV screening landscape varies across the United States (Figure 2). Although CMV is more prevalent than many conditions included in newborn screening programs, only a few states in the country (Texas, Minnesota, and New York) have adopted universal cCMV screening policies to date [7]. In other states, screening follows international recommendations and is often targeted based on hospital or state policies, with testing primarily conducted in infants presenting specific clinical indicators, such as failed hearing screening, microcephaly, a low birth weight, and thrombocytopenia [7]. Despite the variation in current screening protocols, a recent study performed in the US confirmed that the implementation of state newborn hearing screening programs corresponded to increasing diagnosis rates of cCMV with hearing loss (0.87 [0.51–1.22], *p* < 0.001) [52]. Additionally, there were significant increases in early CMV detection in both symptomatic (0.68 [0.37–0.99], *p* < 0.001) and asymptomatic (0.18 [0.03–0.32], *p* = 0.02) neonates [52]. Notably, this increase in diagnosis rates could also be attributed to an increase in education and awareness about this impactful congenital infection.

While newborn hearing screening has significantly improved early CMV detection, those positive for infection but failing to present with the “typical” clinical symptoms may be undetected, delaying crucial intervention.

### 6.2. Around the World

Richard et al. conducted a study in France to characterize the implementation of universal screening for cCMV in neonates via a CMV PCR saliva test, ultimately testing 98.1% of infants in the study period, and they found an infection prevalence of 0.4%; this investigation also found that screening had a high parental acceptance rate and that testing administration was feasible [6]. Further, only 38% of newborns presented with signs characteristic of infection, supporting the notion that, in the absence of universal screening, most cases would remain undiagnosed [6].

As previously discussed, another option for congenital CMV detection is utilizing materials that would otherwise be discarded with DBS screening. In a recent study of 433 infants that assessed the utility of discarded umbilical cord blood, 2 (0.5%) tested positive for CMV in their cord blood, with 1 showing symptoms; both infants had mothers who previously had CMV but showed no recent infection [53].

The Tuscany region of Italy has seen some success with the implementation of universal cCMV screening protocols. Coupling TEOAE and AABR screening with auditory follow-up may positively improve rehabilitation results in terms of hearing abilities and linguistic and communicative skills [54].

In England, targeted surveillance has traditionally been utilized as the primary method for monitoring children who may be at risk of developing hearing loss, regardless of the hearing screening outcome [55]. Currently, the JCIH (Joint Committee on Infant Hearing) maintains the recommendation of monitoring risk factors to detect postnatal hearing loss by 3 months in cases of CMV [56]. Although it is important to diagnose cCMV-associated hearing loss and monitor these children, multiple follow-up visits for monitoring can place excessive strain on families and audiology services [55]. This could leave an opportunity for universal screening to help bridge the gap.

In Japan, a recent investigation utilized PCR screening of urine from 23, 368 newborns to screen for cCMV infection. In addition to the PCR analysis, newborn hearing screening (automated auditory brainstem response (AABR) testing) was conducted within 5 days of birth to examine the incidence of cCMV infection and SNHL, respectively [56]. The study determined that the incidence of late-onset SNHL and neurodevelopmental disorders is associated with and may be predicted by an increase in urinary CMV viral load [57]. This investigation helps support the idea that quantitative viral load measurements may help to more accurately detect the incidence of cCMV infection.

A recent study performed in Australia examined parental opinions on a targeted saliva swab for cCMV from 2019 to 2020 as authorities considered the option of placing screening protocols in the hands of parents [38]. Although the results found that the swab offered a non-invasive screening technique that was generally well received by parents and easily delivered, a high false-positive rate could increase anxiety and uneasiness [38]. This could potentially be mitigated by increasing public knowledge of cCMV and completing the swab at the hospital to reduce the risk of false positives from completing the swab incorrectly or breastmilk contamination [38]. Other studies support the potential for saliva PCR testing to be used in screening protocols as a feasible and effective method, whether that is with the assistance of parental or healthcare worker administration [57].

Countries like Norway have created registries to collect data and set quality standards for newborn hearing screening and early intervention [58]. These registries track key metrics, including the rate of false positives in hearing tests, CMV testing within three weeks for infants who fail screening, the confirmation of hearing status by three months, and the start of intervention within three months of diagnosis [59]. Having registries such as these could help identify the most suitable screening methodology that could be adopted and implemented worldwide.

In Spain, the Commission for the Early Detection of Hearing Loss (CODEPEH) has encouraged universal neonatal hearing screening [59]. This approach helps prevent false negatives in individuals who do not have risk factors for congenital hearing loss, which accounts for 60% of cases [59]. This screening may utilize objective methods such as otoacoustic emissions or automatic auditory evoked potentials (AEPs) and allow for the subsequent testing of cCMV by a PCR of urine and saliva, as well as the opportunity to deliver life-altering treatment [59].

A study based in Germany screened 6102 newborns for cCMV using buccal swabs within the first three days of life, with confirmatory testing for those who tested positive, while ensuring measures to prevent false positives from breast milk [60]. This research highlights a highly effective cCMV screening strategy using eNAT™ medium and quantitative PCR, supporting centralized pooled saliva testing while emphasizing the need for clinical follow-up to establish a viral load threshold for negligible late-onset CMV disease risk [60].

Some studies have investigated the efficacy of establishing universal cCMV testing in premature infants born before 33 weeks of gestation. Out of 549 urine samples/infants identified, none were positive for CMV DNA, suggesting that a combination of failed hearing tests with universal screening could more closely select for and identify those positive for cCMV that may benefit from appropriate treatment [61,62].

In resource-limited geographic locations, there have been some questions regarding the feasibility of implementing universal screening in an already resource-strained landscape. However, studies in Northern India offer positive reassurance that this is possible, with study findings demonstrating the feasibility and value of simultaneous newborn screening for both cCMV and hearing loss in a resource-limited setting [63].

## 7. Discussion

Regardless of screening protocols, the implementation of regular, strategic follow-up plans remains crucial for ensuring timely intervention and optimal outcomes for affected infants [64,65]. Consistent monitoring can help identify late-onset complications and enable early therapeutic measures, ultimately improving long-term prognoses.

Two expert groups, the International Congenital Cytomegalovirus Recommendations Group (ICCRG) and the European Society for Pediatric Infectious Diseases (ESPID), strongly advocate for antiviral therapy in newborns with moderate-to-severe symptomatic congenital CMV (cCMV), based on robust evidence from randomized controlled trials (RCTs) [66]. This treatment approach has been shown to mitigate short-term and potentially long-term hearing deterioration, reinforcing its role in clinical management. However, a significant challenge lies in the fact that the majority of cCMV cases present mild or subclinical manifestations, including isolated sensorineural hearing loss (SNHL), which often remain undetected in the absence of targeted screening [59,67]. As a result, treatment recommendations vary across guidelines [58], reflecting the ongoing debate regarding the necessity and efficacy of antiviral therapy in these cases.

Despite these uncertainties, the benefits of early detection and intervention, such as hearing amplification, speech therapy, and other supportive measures, are increasingly evident for children at risk of late-onset SNHL. Universal screening programs play a pivotal role in identifying these at-risk infants, allowing for timely intervention that may significantly enhance developmental and auditory outcomes. A universal hearing screening protocol that can be easily integrated into routine newborn screening labs would be ideal and perhaps offer the most consistent rates of early cCMV detection, as shown in recent pilot programs [68]. Integrating CMV diagnostics into routine newborn screening workflows may not only substantially increase detection rates but also ensure timely care pathways in an interdisciplinary manner and a multifactorial approach.

Looking forward, the future of cCMV diagnostics is braced for scientific, medical, and technological advancement. First, the development of rapid, high-sensitivity molecular assays that can be seamlessly integrated into existing newborn screening platforms will revolutionize the means of cCMV detection. Rapid PCR analyses of saliva and dried blood spots have previously been studied in terms of their utility for early detection, offering success while also identifying areas for improvement. It will remain important that the method(s) utilized for early cCMV detection are implemented as low-cost, efficient options with high reproducibility and specificity. Additionally, as artificial learning algorithms continue to become intertwined within the field of healthcare, they may be used to assist the diagnosing and managing clinician. With the potential to identify subtle clinical patterns, enhance risk stratification, support individualized follow-up plans, and connect families with specific resources, this may act as a key resource in maximizing early cCMV detection and optimizing outcomes. Further, continued investigation into the human genome could be important for genomic and proteomic profiling that could offer key clues to novel biomarkers that may be used for the early detection and ultimate prognosis of complications related to cCMV.

The continued sharing of trials related to diagnostic methods will continue to be useful in shaping consistent screening and treatment practices across healthcare systems worldwide. These advancements, combined with continued advocacy and public health investment, have the potential to transform cCMV from a frequently missed diagnosis into a condition that is systematically identified, monitored, and managed from the earliest days of life.

In sum, a comprehensive approach that combines universal screening with structured follow-up and evolving diagnostic technologies is essential for optimizing the care and outcomes of infants with cCMV. Future advances in diagnostics and individualized therapeutic models offer a promising outlook, working to close the current gaps in detection and treatment and improving the quality of life of affected children and their families.

## 8. Conclusions

Because there is no effective CMV vaccine available to date, congenital cytomegalovirus remains a significant global health burden, particularly affecting children of mothers from lower socioeconomic backgrounds. Although most cases of cCMV are asymptomatic, nearly 25% of infants will go on to develop adverse neurodevelopmental outcomes, including sensorineural hearing loss; vision impairment; and speech, language, and learning delays. Apart from educating women on how to reduce their risk of contracting CMV during pregnancy, CMV screening programs are currently the best preventative care measure in improving the outcomes of children with CMV, as they allow for early detection and, more importantly, timely intervention with antiviral therapy. Targeted screening is currently the standard of care due to its practicality and feasibility. However, in the future, and if infrastructure improves, we may expect more states and countries to adopt universal CMV screening programs to identify all those with CMV infections, including infants who would develop hearing loss later in life.

## 9. Future Directions

Future policy and research initiatives must aim to improve universal CMV screening programs to detect and intervene early for all such affected infants, including those at risk of late-onset disease. Although targeted screening is the present norm because of its practicality, advancements in technology and healthcare infrastructure may, in the future, make universal screening a more functional option. Large-scale comparisons of the cost-effectiveness and long-term efficacy of universal screening will be most important to guide policy changes and broader implementation. Refining the diagnostic tools to make them more accurate and efficient at screening, such as point-of-care testing or newborn screening panel tests, also has the potential to integrate early detection into streamlined procedures.

In parallel, continued investment in vaccine research remains a critical priority in the fight against congenital CMV. The development of an effective CMV vaccine could significantly reduce the incidence of congenital infections and alleviate the long-term neurodevelopmental burden associated with the virus. Ongoing clinical trials exploring potential vaccine candidates must be supported through sustained funding and global collaboration to accelerate progress. Furthermore, expanding public health initiatives to educate women of childbearing age and families on CMV transmission prevention strategies remains essential. Combining enhanced screening programs, vaccine development, and widespread education efforts can create a comprehensive approach to mitigating the impact of congenital CMV on a global scale.

## Data Availability

No new data were created or analyzed in this study. Data sharing does not apply to this article.
